# Holding it all together: Family caregivers’ support needs after very early supported discharge post stroke

**DOI:** 10.1371/journal.pone.0345795

**Published:** 2026-03-26

**Authors:** Karin Törnbom, Gunnel E. Carlsson, Katharina S. Sunnerhagen

**Affiliations:** 1 Institute of Neuroscience and Physiology, Department of Clinical Neuroscience/Rehabilitation Medicine, Sahlgrenska Academy University of Gothenburg, Gothenburg, Sweden; 2 Department of Social Work, University of Gothenburg, Gothenburg, Sweden; 3 Department of Public Health and Community Medicine, Institute of Medicine, Sahlgrenska Academy, University of Gothenburg, Gothenburg, Sweden; 4 Rehabilitation Medicine, Sahlgrenska University Hospital, Gothenburg, Sweden; Duervation, AUSTRIA

## Abstract

**Background:**

Early supported discharge (ESD) facilitates the transition from specialized stroke units to home-based rehabilitation in familiar environments. While ESD has shown positive outcomes for people with stroke, little is known about how their family caregivers experience this transition and the support available once ESD ends. This qualitative study explores the needs, challenges, and expectations of family caregivers during the period following very early supported discharge (VESD) in Sweden.

**Methods:**

In-depth, individual, semi-structured interviews were conducted with twelve family caregivers of persons who had received VESD after stroke. Data were analysed using thematic analysis with an inductive approach. This design was chosen to capture the caregivers’ rich experiences and to allow themes to emerge directly from the data.

**Results:**

The findings showed that while the VESD intervention provided important initial support, its limited duration left family caregivers feeling unprepared, anxious, and overwhelmed by their new responsibilities. The overarching themes indicated that caregivers faced emotional strain alongside new and unclear demands. Many struggled to balance caregiving with work and family life, while also coping with relationship changes. The caregivers perceived their role as informal medical coordinators and advocates for the person with stroke within the healthcare system to be particularly challenging. The absence of structured follow-ups, professional guidance, and caregiver-focused support further intensified these challenges.

**Conclusions:**

This study underscores the need for sustained and comprehensive long-term strategies that include education and mental health support for caregivers in the rehabilitation process after stroke. Moreover, the integration of caregivers into rehabilitation is essential to ensure their preparedness and well-being. Such measures are crucial not only for reducing caregiver burden, but also for promoting better long-term outcomes for both caregivers and the person with stroke.

## Introduction

Stroke remains one of the leading causes of long-term disability worldwide, placing a significant burden not only on the person with stroke, but also on their family caregivers [1,2]. The caregiving role, often assumed by family members, is associated with physical, psychological, and economic burdens, as caregivers often navigate the demands of rehabilitation, daily care, and emotional support for their loved ones [1–4]. Previous studies have established that the severity of stroke symptoms and the common subsequent dependency, directly correlate with an increased caregiver burden, triggering stress, anxiety, and depression [5,6]. Furthermore, research has defined the emotional burden on stroke caregivers, emphasizing the social and psychological challenges that stem from witnessing the personality changes and gradual decline, which can compromise not only the patient’s recovery but also the caregiver’s quality of life [7,8]. In addition to the immediate practical challenges, the long-term nature of stroke care contributes to a strain on caregivers. This cumulative burden is exacerbated by a lack of sufficient external support and inadequate coping resources, which leaves many caregivers feeling isolated and overwhelmed [9].

In recognition of all challenges after stroke, many healthcare systems have adopted home-based interventions, so called early supported discharge (ESD) teams, to facilitate rehabilitation and the homecoming process, with the aim of supporting both patient recovery and easing caregiver burdens [10–12]. One such intervention is the very early supported discharge (VESD) team, which provides short-term, intensive rehabilitation and professional support at home following discharge from a stroke unit [13]. Although the primary intent of this service is to optimize the immediate post-stroke recovery from the patients’ goal settings [14], it also plays a crucial role in shaping the caregiving experience in the early phase after stroke [15].

Previous research has highlighted that the transition from hospital to home is often fraught with uncertainty, stress, and an overwhelming sense of responsibility among family caregivers [16,17]. While caregivers frequently express gratitude for the structured and continuous support provided by interdisciplinary ESD teams during this critical period, gaps in emotional and informational support, especially for those balancing caregiving duties with personal employment or own health concerns, can leave caregivers feeling isolated once the formal intervention ends [18–20]. In addition, emerging evidence suggests that the benefits of a consistent and professional long lasting relationship are instrumental in building trust and fostering effective communication, which in turn enables caregivers to navigate daily challenges with greater assurance [19,21].

The present study investigated the multifaceted experiences of family caregivers involved in post-discharge multidisciplinary interventions provided by the VESD team in Sweden. This study aims to explore how caregivers adapt to the dual demands of managing the long-term physical and psychological consequences of the disease, alongside their evolving caregiving roles. Hence, this study aimed to explore family caregivers’ experiences of providing care at home for a close family member with stroke after the Very Early Supported Discharge (VESD) team’s home-based intervention was ended.

## Materials and methods

### Study design

This study is an interview study nested in a randomized controlled trial, GOTVED [22], comparing VESD, including a home-based rehabilitation intervention from a coordinated multidisciplinary team, with conventional care after stroke. To obtain a deeper understanding of how very early supported discharge combined with rehabilitation is given to persons with stroke and their caregivers, we adopted an interpretivist standpoint [23]. The epistemological position is subjectivism, and the ontological position is relativism, where reality is seen as subjective and varies from person to person [23]. A further standpoint was based on interactionism, where people are seen as actors creating their own reality through interaction with the social world [24,25].

While ESD is the term used in the literature on early supported discharge [26], the term used in the GOTVED study and in the current study is very early supported discharge (VESD), where the added “V” specifies that the discharge from hospital to home took place very early after the stroke as compared with previous studies of ESD. The time point for discharge was determined according to the patient’s medical condition. For more information about how the VESD intervention was constructed, the study protocol is referred to [27] as well as the S3 File.

The study was approved by the Regional Ethics Committee of the Western Region of Sweden and written informed consent was obtained from all participants (identification numbers 110221, 042–11).

### Participants

Individuals sharing a household with participants in the GOTVED intervention group (n = 69) were consecutively recruited [22]. The criteria for inclusion of persons with stroke were specific, consisting of the following strategy: confirmed stroke according to the WHO criteria, > 18 years of age, living within 30 minutes from the stroke unit and a MoCA index < 26 if BI = 100 on day 2, 0–16 points according to the National Institute of Health Stroke scale (NIHSS) and BI 50–100 points. The exclusion criteria were not being discharged home, NIHSS >16, BI < 50, life expectancy < 1 year (as with severe malignancy), or not speaking or communicating in Swedish prior to the incidence [22].

Nineteen people sharing a household with patients randomized to the VESD and home rehabilitation after stroke, that is to the intervention group in GOTVED, were consecutively informed about this study by a person who was not involved in this part of the GOTVED. Of the 19 caregivers available, all consented verbally and in written form to be interviewed. Three participants were excluded prior to the interview: two declined participation due to tiredness, and one was not sufficiently fluent in Swedish; no professional interpreter was available. Furthermore, four caregivers could not participate due to logistical reasons, which resulted in a study population of 12 participants. Of these, nine were females and three males. Saturation was defined as the point at which no new themes or meaningful variations emerged in the interviews. After conducting twelve interviews, the research team assessed that additional interviews were unlikely to contribute substantially new insights in relation to the study aim [28].

At inclusion on day two after stroke onset all persons with stroke were considered to have mild stroke with an NIHSS score of 0–4. The median length of hospital care in the stroke unit for patients with stroke was 9 days. The VESD intervention was delivered 2–4 times/week (over four weeks) in the patient’s home. The interviews were conducted at a median time elapsed since discharge from the hospital of three months and 1–2.5 months after the VESD had ended.

The participants’ demographic and clinical characteristics are shown in **Table 1**.

**Table 1:** Characteristics of persons with stroke and their family caregivers.

Int nr	Person with stroke age at admission	Caregivers age at admission	Caregivers work at stroke onset	Caregivers living with the person with stroke	Caregivers work at follow up	Caregivers Sex	Days at stroke unit	Stroke severity at inclusion NIHSS (0–42)	Barthel Index at inclusion (0-100)	MoCA at inclusion (0-30)
1	45	44	Yes	Yes	Yes	M	8	4	90	–
2	71	68	Yes	Yes	Yes	F	10	0	90	25
3	51	59	Yes	Yes	Yes	F	9	0	100	29
4	81	79	No	Yes	No	F	11	0	70	24
5	83	67	Yes	Yes	Yes	F	8	3	75	19
6	68	68	No	Yes	No	F	17	3	85	15
7	65	63	Yes	Yes	Yes	F	21	3	65	27
8	73	69	No	Yes	No	F	5	0	60	25
9	78	80	No	Yes	No	M	6	0	90	25
10	68	67	Yes	Yes	Yes	F	10	2	100	19
11	81	76	No	Yes	No	F	5	0	85	27
12	82	68	No	Yes	No	M	14	1	55	21

All participants are hereafter referred to as “caregivers” or “family caregivers.”

### Data collection

The interview data were collected by ÅN, a physiotherapist with >4 years of clinical experience in stroke rehabilitation and PhD student, under the supervision of ÅBA, a nurse and associate professor with >15 years’ experience in qualitative research. There was no relationship between the interviewer and the participants prior to the study period. The participants were interviewed once, approximately three months after the patient was discharged from the hospital to their shared home. The caregivers were free to decide where they preferred the interview to take place. Accordingly, nine of the interviews took place in the participants’ homes, two at their workplaces and one in a secluded room at the research unit. The interviews were conducted without the patient being present in the room. To avoid losing important information, all interviews were audio-recorded and transcribed verbatim.

An interview guide (ÅN, KSS) with open-ended questions about the caregivers’ experiences of the intervention, and of managing everyday life in the context of very early supported discharge was used comprising 1) The caregivers’ experiences of the patient coming home and being at home and 2) the caregivers’ experiences of the support and rehabilitation at home from the VESD team. To enrich the data, follow-up questions were asked that were specifically tailored to each answer. The interview guide appears in its complete form in both English and Swedish (S1 and S2 Files).

### Data analysis

Data were analyzed by two researchers KT, a social worker, PhD, experienced in qualitative methods and with ten years of experience in stroke rehabilitation research, and GC, OT, PhD, a senior researcher with more than twenty years of experience in qualitative research and 40 years of experience in stroke rehabilitation. All data interview procedures were carried out between 1 September and 28 January 2025.

Inductive thematic analysis was performed, as described by Braun and Clarke [29]. This approach was used to identify, analyze, and report patterns within data, aiming to organize the data into patterns of semantic content and further to transform data into an interpretative level. Authors could not identify participants during this stage.

The interviews were read repeatedly by the authors/researchers GC and KT to become familiarized with the data, and initial ideas about the content and interesting features of the data were extracted. Systematic coding of the entire dataset generated initial codes conveying relevant information in relation to the research question. The codes were combined into potential subthemes and themes based on how the different codes were related. To strengthen the trustworthiness of the results throughout the process regular meetings were held by the researchers GC and KT to compare and discuss the analyses, and discrepancies were discussed until a consensus was reached. Thereafter, the analyses were reviewed by the third researcher and author (KSS), and the relevance of the codes, subthemes, and themes were checked and discussed. Discrepancies between the three authors were discussed until a consensus was reached. The analysis process involved moving back and forth between the entire dataset and data extracts and between the different steps in the analysis. The final manuscript was reviewed and approved by all the researchers involved. Participant validation was not undertaken, as participants were in an emotionally demanding situation and additional contact was considered burdensome. Participants were informed that they were welcome to read the published article if they wished. We acknowledge that the absence of formal member checking may be regarded as a limitation in terms of credibility [30].

## Results

The analysis resulted in a framework with the following themes: 1) How the family caregivers experienced the VESD team’s intervention, 2) The initial period after the VESD team concluded their support, 3) Some caregivers were greatly affected by personality changes in the person with stroke, 4) The family caregivers felt alone with an overwhelming sense of responsibility, 5) A lack of long-term support and guidance. All themes were inherently exclusive, and their contents are described below. The results are presented using illustrative quotes, and all participants’ names have been anonymized.

### How the family caregivers experienced the VESD team’s intervention

The families were very grateful for the efforts of the VESD team and felt that they would not have been able to cope without this help and support. The first time at home was described as very shaky and insecure and being able to lean against a rehabilitation team was described as invaluable. For those who were employed, the intervention allowed them to return to work, which was important for the economy, while feeling reassured that the person with stroke was left in safe hands.

The caregivers also emphasized the importance of allowing the person with stroke to undergo rehabilitation in their home environment, which was described as contributing to a greater sense of security and well-being for the patients. Caregivers and persons with stroke greatly appreciated the team’s ability to answer questions, provide referrals when necessary, and suggest solutions to various challenges. Additionally, having continuous contact with the same healthcare professional was considered valuable, as it allowed the affected individual to build relationships with their formal caregivers.

“The fact that it is the same people coming each time, I think that is really positive because it allows for a personal relationship, which I believe is very important. You feel more comfortable asking questions. And you don’t have to answer the same questions over and over again, which makes things easier.” (Caregiver Stig, id 12)

The caregivers’ personal contact with the VESD team varied. Retired caregivers were more present when the team visited, allowing them to express their concerns and ask questions during the visit. In contrast, working caregivers had little to no direct interaction with the VESD team. The latter group also felt that they had not received any support or assistance. They expressed that they had been given very little information, making it challenging when they were later left alone to care for the person with stroke.

“But no one really asked me how I was doing, and I completely understand that… they don’t have time for the relative too. So I had to process it as best I could. I didn’t talk much with the staff, and I didn’t receive any support. But I am quite a strong person… even though it was a traumatic experience for me… but I picked up a lot of good brochures at the ward, and I know that I can turn to the Stroke Forum if needed.” (Caregiver Sonja, id 8)

Other caregivers more explicitly expressed that they had needed emotional support for themselves during the initial period and that the lack of such support had been difficult.

### The initial period after the VESD team concluded their support

Family caregivers described feeling alone and abandoned when the team’s support ended after 1–4 weeks. Regardless of the intervention duration, all caregivers expressed a wish for it to last longer. Initially, there was significant anxiety that the person with stroke might fall ill again, leading them to monitor the patient closely in daily life. This could involve listening to their breathing at night or following them during the day to prevent falling. They were also careful to ensure that the person with stroke rested regularly and did not become overly fatigued, as advised by the VESD team.

“I still felt that I had to make sure he didn’t eat too much, and didn’t do anything crazy... but he became very irritated when I told him, and he’s stubborn, so now I try not to do that anymore. I stay out of the way. But in the beginning, of course, I was shocked.” (Caregiver Sonja, id 8)

This initial period alone at home with the person with stroke was described as being highly stressful. Some caregivers experienced significant sleep disturbances and found it difficult to relax due to their worries. They often felt a strong need to stay at home or ensure that a family member was present to monitor their loved ones. At the same time, there was a great deal of new information to learn and adapt to, which added to their sense of worry and tension.

“There was so much in the beginning, with medications and just making sure he stayed alive... I would have liked another doctor’s appointment at that point, after the team had left, so that I could ask my questions. It would have made things a lot easier.” (Caregiver Ann-Britt, id 10)

After a little longer at home, a new concern was established, which was more about securing the person with stroke from injuring himself or making decisions that could be wrong or destructive. The family members described how they gradually became aware of the everyday situations that no longer functioned properly and how the person with stroke’s limited insight into their illness, combined with a strong desire to return to life as it had been before the stroke, created significant difficulties in daily life.

“He has no insight... and sometimes I have to scold him because he doesn’t understand. And then he thinks I’m treating him like someone with an intellectual disability. But I’m not, because he wouldn’t have done those things if he hadn’t had the stroke. And I have to react in a way that makes him respond. It’s just like with children... first, I have to stop him, and then I can explain what’s wrong.” (Caregiver Lisbeth, id 7)

### Some caregivers were greatly affected by personality changes in the person with stroke

Several caregivers described how the persons with stroke underwent a change in personality, becoming more easily irritated and angry than before the stroke. In daily life, family caregivers shouldered the frustration and anger when things did not unfold as the person with stroke expected. At times, family caregivers were verbally attacked and called hurtful names, and some family members explained that it was important to maintain a distance when the affected person was in a bad mood. Some caregivers’ had considered getting a divorce or leaving the person with stroke:

“I notice that I can’t tell him off, because then he gets angry, so I have to be careful about that. He’s not a rude person really, but he yells at me now, even though it’s not my fault. It’s a big difference compared to how he was before. It’s like he wants to bring me down when he can’t manage things himself… But you can’t leave someone who is sick, can you do that? (Caregiver Marie, id 5)

Several described the person with stroke as “a kind person,” but noted that the stroke had changed them significantly. Caregivers explained how they became cautious about saying the wrong things and avoided pointing out when something went wrong. These avoidance and conciliating strategies were used to prevent conflicts and arguments in daily life. Caregivers did not want to be yelled at and wanted their everyday lives to proceed as smoothly as possible. In many situations, it was put forth as a relief when professionals intervened and set boundaries so that family caregivers did not have to take on the conflict. This could include issues such as driving licenses, shooting, and other leisure activities.

“His judgment fails him, so he could go and fetch the balls when others were hitting, and... then it was the instructor’s problem, not mine, so the instructor had to yell at him, and at least then he was angry with the instructor, not with me.” (Caregiver Lisbeth, id 7)

Another strategy was to assign tasks to the person with stroke that “couldn’t go too wrong.” This included tasks such as mowing the lawn or vacuuming, but not repainting the house, and there were family caregivers who hid car keys and bills. They also selected tasks that could be forgotten without major consequences; “I might ask him to start the washing machine, and if he forgets, I can do it myself when I get home.”

Other participants did not notice any significant behavioral changes and described an easier return to their daily lives. In these cases, fatigue was often a significant issue. Participants observed that they had to adapt to a new way of living, where everyday tasks were allowed to take longer, and they did fewer activities in their free time. Middle-aged couples were perhaps most affected by this change in activity levels, as they stopped or significantly reduced socializing and engaging in leisure activities. Some family caregivers felt very restricted and trapped in their role as constant caregivers;

“Yes, we probably won’t be able to sail anymore, because Nils has no balance... and we can’t travel like we used to... and I don’t leave the house anymore because he doesn’t want me to go anywhere, I don’t go to the gym, I don’t go for walks, I don’t meet my friends... none of that. And he wants me to listen to him all the time, and he only talks about his illness… and he talks about it all day long, now on Saturday and Sunday, he has talked about this almost the entire time.” (Caregiver Johanna, id 3)

Caregivers mentioned that they were now simply grateful for the occasional opportunity to get away to the countryside or meet up with a few friends. Life was described as much more monotonous and difficult compared to before the stroke, and some felt that nothing seemed enjoyable anymore. Family caregivers of younger age also described the additional burden of balancing their professional work, managing all household responsibilities, and taking care of the children.

“I had to take on a much bigger load, it affected my life in that way. First, I worked a lot, then I had to shop, do the dishes, change light bulbs, take the recycling, everything that needed to be done. We used to share the responsibilities. So, I felt that if I do the laundry, you can hang it up. But he lost a lot of initiative there.” (Caregiver Ylva, id 6)

### The family caregivers felt alone with an overwhelming sense of responsibility

All the worry and increased responsibility made the caregivers feel alone. Caregivers felt that the healthcare system did not listen to them, that their concerns were not taken seriously, and that they were shouldering too much responsibility alone. Some family caregivers called for scheduled follow-up meetings after the VESD intervention, with some meetings specifically focusing on the needs of the family member. It was sometimes seen as a problem that the person with stroke presented everything so positively to the healthcare professionals, and that they often seemed to lack insight into their illness;

“Yes, according to him, there are no problems, everything is great and progressing. And there are no problems when anyone asks him. Which can be very frustrating for me, as I see the whole picture.” (Caregiver Anna, id 2)

Several caregivers also described feeling uncertain about where to seek help. Primary healthcare providers were not very familiar with stroke and could not answer their questions, while specialists were extremely difficult to reach.

Some caregivers were also concerned about medical aspects. One caregiver described how her family member had been prescribed two incompatible medications, and she had to bring this to the doctor’s attention. After this incident, she developed a sense of worry and felt compelled to research every new medication on the FASS (Sweden’s official drug compendium) website. There was also a concern that the person with stroke might forget to take or refuse their medication, and this worry was more widespread among caregivers.

In this isolation and vulnerability, stress and anxious thoughts increased, while some speculations and assumptions about stroke and rehabilitation took root in their minds. One caregiver feared that the stroke would only worsen over time, while another believed that all symptoms and impairments would completely disappear after six months. One caregiver thought that stroke often led to persistent depression and therefore did not seek help for her husband’s low mood, simply thinking that it was not worth it.

“Doesn’t the stroke just keep getting worse? And worse? Like, it’s only going downhill? I feel like he’s getting more and more irritable… At first, when he got home, he was happier and more positive. But now, it’s worse, much worse.” (Caregiver Marie, id 5)

The perceived lack of contact with healthcare providers thus not only created anxiety but also led participants to form their own perceptions and ideas about the disease and its progression. During the interview, it was clear that the family members “took the opportunity” to ask the interviewer various questions about rehabilitation, the course of the illness, the possibility of improvement, and the risk of recurrence – questions they would have liked to ask professional stroke experts.

### A lack of long-term support and guidance

Family caregivers expressed a need for more guidance and support, both for themselves and in the form of long-term follow-ups for the person with the stroke. Several caregivers reported that the person with stroke had not received any follow-up regarding their medications and that healthcare services had not been in contact at all after the initial three months. They requested an evaluation from healthcare professionals to better understand the progress of rehabilitation, as they currently had to guess which abilities had improved.

“My husband is so positive and says everything is fine... so for my part, to really know, a follow-up visit is necessary. Sometime after four months, I would like to be present and hear the assessment—how he is doing, whether he can drive a car, and so on.” (Caregiver Anna, id 2)

Family caregivers also described how they had not received any support from the healthcare system, neither from the VESD team nor afterward. Some explained that their anxiety and concerns had not subsided, even though a significant amount of time had passed since their stroke. They suggested that more opportunities to meet and speak with healthcare professionals could provide reassurance, both to get answers to their questions and to receive emotional support. At the same time, they acknowledged that the healthcare system had limited resources and understood that they would have to process their experience largely on their own.

For those who were struggling with their own health issues, the lack of support was particularly difficult:

“This has been very distressing for me as well… you have to be available all the time. And I haven’t been well either—I have severe problems with my back and feet, and when you are unwell yourself, it limits what you can do. I also get migraines when I’m overexerted, when it all becomes too much… But you more or less become a constant caregiver.” (Caregiver Sofie, id 4)

Family caregivers also described how challenging it was when relatives and friends did not understand the true extent of the affected person’s condition. They felt that no one recognized how much effort they put in every day, as the person with stroke “looked so healthy.” Some also noted that the person with stroke seemed livelier and more engaged when visitors were present but acted differently once they had left. Meaning that it was exhausting for family caregivers that those around them did not see how much they had to adapt to cognitive impairments, mood changes, and, in many cases, the person with stroke’s lack of awareness about their own condition.

## Discussion

The main purpose of this article was to explore how family caregivers experienced taking care of their close relative with stroke after the VESD-team had concluded its intervention. After the intervention ended, caregivers played a crucial role in their caregiver’s recovery process and in navigating how to build the best possible life together after stroke. However, caregivers felt that they lacked the resources, knowledge, and sufficient support to shoulder the responsibility as primary caregivers.

Caregivers in this study felt that the VESD intervention was supportive but had ended too quickly. They didn’t feel prepared to take care of the person with stroke after only 2–3 weeks at home. Caregivers reported feelings of being overly anxious and overwhelmed, as they quickly had to adjust to providing care, while facing their own personal emotional challenges connected to their caregiver’s stroke. As described previously, caregivers often experience unpreparedness and practical as well as emotional challenges during the first time alone with the person with stroke [31–33]. In the current study these challenges were most pronounced among working caregivers. They found it difficult to balance their roles as caregivers, employee and carer for home and sometimes young children. Further, they experienced a sense of loss, grieving the relationship and pre-stroke life they had with their partner. Previous research showed that many caregivers face a crisis when taking responsibility for the patient’s well-being and managing new tasks during this challenging first time at home [34].

Feelings of being left to manage alone with a huge responsibility was pronounced among most caregivers. They also experienced an insufficient professional support and guidance, which is well known from previous research [35–39]. Previous research further suggest that caregivers to persons with stroke should be involved in rehabilitation, by improving continuity of care between specialist and generalist services, and that caregivers need support in their own emotional and role management [35,39]. Such support was lacking in the present study, as caregivers reported that primary healthcare was unable to address their questions and that medical and rehabilitation expertise from the stroke unit was unavailable following the conclusion of the VESD intervention.

This sense of unavailable support, in turn, heightened caregivers’ feelings of anxiety, inadequacy, and an overwhelming sense of personal responsibility. Previous findings underline that caregivers do not have time to deal with the shock and crisis of the stroke event, nor the crisis of discharge and all of their new responsibilities [38]. A mixed-methods study found that the absence of structured support systems, such as respite care or community resources, left many caregivers feeling overwhelmed or even burned out, having a significant negative impact on the person with stroke’s recovery outcomes [40]. In our study, this was expressed through participants’ worry and concerns about where to direct their questions. Caregivers felt that life had become overwhelmingly burdened with responsibility, while the joy and leisure they experienced before stroke were noticeably absent. Based on our findings and previous research, there appears to be a significant need for improved support systems and caregiver education to enhance their well-being and the quality of care they provide [41].

Caregivers in our study highlighted issues regarding the person with stroke’s personality changes and how these impacted their relationship and shared life. Several caregivers felt the need to watch over and look after the person with stroke, while simultaneously doing so without the person feeling monitored or treated like a child. Caregivers explained how they sometimes walked on eggshells to avoid situations where the person with stroke became angry or irritated and would snap at them. These challenges are often linked to the use of negative coping strategies such as escape-avoidance and distancing [42] and may affect the caregiver’s ability to empathize and collaborate effectively with the person with stroke [43]. Earlier research argued that caregivers who are yelled at or demeaned by the person with stroke, leads to hurt feelings and ultimately dysfunctional relationships [7,43–45]. Recent research showed that family members often struggle to communicate effectively with the affected individual, leading to misunderstandings and feelings of being a burden to each other [46]. Caregivers in our study removed dangerous items and assigned the person simpler tasks that they could manage, to avoid discussions and conflicts. These strategies were used to take control of the situation, while simultaneously aiming to give the person with stroke a false sense being in control. The findings suggest a need for improved communication and structured support systems specifically targeting caregivers. In the absence of such support, caregivers may be left feeling unsupported and insufficiently prepared to manage personality changes in the person with stroke [39].

Another similar difficulty that caregivers brought up as a problem was how they would prevent the person with stroke from doing something potentially dangerous. For several caregivers this was a huge worry in daily life. It was previously concluded that caregivers who accept and get used to personality changes in their caregiver, and who develop effective coping strategies feel better [42,47] This was indicated in our study, but of course easier said than done among caregivers to caregivers at high risk of injuring themselves or others. A constant worry in monitoring a potentially unpredictable or dangerous person with stroke impacts the caregiver’s psychological well-being [48,49]. In addition, previous research concluded that while caregivers often develop strategies to ensure safety, the emotional toll in doing so can be significant [50]. There is a need to improve interventions like support groups, and family-focused interventions to involve the caregiver and the person with stroke, both to improve their coping skills and to reduce caregiver burden [51–53]. Addressing caregiver’s mental health needs is vital to sustain their ability to provide care [50], and to sustain a healthy relationship considering that several caregivers in our study felt so emotionally drained that they considered a divorce.

Several caregivers in the present study reported limited knowledge about stroke and stroke rehabilitation. This was evident in how caregivers took the opportunity to ask the interviewer about the progression of the disease, expected deterioration and future prognosis. It was previously found that educational programs tailored to caregivers, significantly improved their understanding of stroke care and rehabilitation needs, improving both their own and their caregiver’s health [54,55]. According to Swedish guidelines, structured follow-ups with caregivers and a strengthened family perspective that requires governance, knowledge, and good working conditions is needed [56]. Despite this, many caregivers experienced that support is minimal after the initial weeks. Tools like the Post Stroke Checklist (PSC) [57] and digital platforms such as Rehabkompassen [58] are used in Sweden to create personalized rehabilitation plans, addressing individual patient needs, however the focus on caregivers is more scarce [59]. To improve the Swedish rehabilitation process for caregivers after stroke, structured more frequent follow-ups, caregiver education programs, and mental health support should be integrated into post-stroke care to ensure caregivers receive the assistance they need.

## Conclusions

In summary, the findings of this study indicate that while the VESD intervention offered essential initial support to family caregivers after a stroke, its short duration left many feeling unprepared and overwhelmed by the transition to full-time caregiving. Caregivers struggled to balance their new responsibilities with personal and professional demands, experiencing heightened anxiety and a profound sense of isolation. The loss of structured follow-up and guidance exacerbated these challenges, underscoring the need for comprehensive, continuous post-discharge care that includes tailored caregiver education, emotionally supportive interventions, and sustained professional support over time. Together, these findings highlight the critical importance of integrating long-term support strategies into stroke rehabilitation to better prepare caregivers for the complex, evolving role they must adopt in the recovery process.

## Supporting information

S1 File Interview guide in English. (DOCX)

S2 File Interview guide in Swedish. (DOCX)

S3 File Information about the VESD intervention. (DOCX)
