# Supplementary material for: Holding it all together: Family caregivers’ support needs after very early supported discharge post stroke
Source: PLoS One. 2026 Mar 26;21(3):e0345795. doi: 10.1371/journal.pone.0345795 (PMC13020782; doi:10.1371/journal.pone.0345795)
Supplement: S1 File — (DOCX) [file pone.0345795.s001.docx]

# Appendix 1. Interview Guide – Family Caregiver Interview

## Purpose of the Interview:

To explore how it is experienced to be a family caregiver to a person with stroke who returns home under the conditions provided by a Very Early Supported Discharge (VESD) and home-based rehabilitation.

## Background Context for Interviewer (not read aloud):

Hospital stays in stroke units have become significantly shorter in recent years, which means that patients may return home relatively soon after the onset of stroke.

## Interview Questions:

### 1. Early Discharge Experience

- How did you experience the fact that [name/your family member] came home as early as [X] days after the stroke?
- What has that meant for you?
- In what ways has this affected you and your life?
- What are your thoughts about the new situation you’ve found yourself in?

### 2. The VESD Intervention

[Name] received support at home from a nurse, an occupational therapist, and a physiotherapist for approximately four weeks.

- What are your thoughts about the support [name] received from the team?
- What aspects did you find helpful or less helpful?
- How would you describe the support provided by the hospital—for both the person with stroke and for yourself?
- What was your contact with the stroke home team like?
- What was it like to have healthcare professionals coming into your home?

### 3. Impact on You

- How has the stroke and everything that followed affected you and your daily life?

## Notes for Interviewer:

- Use follow-up questions to deepen the participant’s reflections (e.g., “Can you tell me more about that?”, “How did that make you feel?”, “What happened then?”).
- Be attentive to emotional cues and offer pauses when needed.
- Use pseudonyms or neutral terms when documenting responses.
