# Supplementary material for: Holding it all together: Family caregivers’ support needs after very early supported discharge post stroke
Source: PLoS One. 2026 Mar 26;21(3):e0345795. doi: 10.1371/journal.pone.0345795 (PMC13020782; doi:10.1371/journal.pone.0345795)
Supplement: S2 File — (DOCX) [file pone.0345795.s002.docx]

# Bilaga 1. Intervjuguide – Intervju med närstående

## Syfte med intervjun:

Att undersöka hur det upplevs att vara närstående till en person med stroke som återvänder hem under de förutsättningar som mycket tidig understödd utskrivning (VESD) och hemrehabilitering innebär.

## Bakgrund för intervjuaren (ej för uppläsning):

Vårdtiderna på strokeenheten har förkortats kraftigt under senare år, vilket innebär att patienter kan komma hem relativt snart efter insjuknandet.

## Intervjufrågor:

### 1. Upplevelse av tidig utskrivning

- Hur upplevde du att det var att [namn/din anhörige] kom hem redan efter [X] dagar?
- Vad har det inneburit för dig?
- Hur har detta påverkat dig och ditt liv?
- Vad tänker du om den nya situation du har hamnat i?

### 2. VESD-insatsen

[Namn] fick stöd i hemmet av en sjuksköterska, arbetsterapeut och fysioterapeut under cirka fyra veckor.

- Vad tycker du om det stöd som [namn] fick av teamet?
- Vilka delar upplevde du som hjälpsamma eller mindre hjälpsamma?
- Hur upplever du att stödet från sjukhuset fungerade – både för den som fått stroke och för dig som närstående?
- Hur såg din kontakt med strokehemteamet ut?
- Hur har det varit att ha vårdpersonal i hemmet?

### 3. Påverkan på dig

- Hur har det som hänt påverkat dig och ditt vardagsliv?

## Observera för intervjuaren:

- Använd följdfrågor för att fördjupa deltagarens reflektioner (t.ex. ”Kan du berätta mer om det?”, ”Hur kändes det?”, ”Vad hände då?”).
- Var uppmärksam på känslomässiga signaler och ge utrymme för pauser vid behov.
- Använd pseudonymer eller neutrala termer vid dokumentation av svar.
