# Supplementary material for: Holding it all together: Family caregivers’ support needs after very early supported discharge post stroke
Source: PLoS One. 2026 Mar 26;21(3):e0345795. doi: 10.1371/journal.pone.0345795 (PMC13020782; doi:10.1371/journal.pone.0345795)
Supplement: S3 File — (DOCX) [file pone.0345795.s003.docx]

The Very Early Supported Discharge (VESD) intervention:

The VESD intervention commenced prior to discharge from the stroke unit with a person-centred and goal setting meeting involving the stroke nurse coordinating the VESD team, relevant health professionals (physiotherapists and occupational therapists), the patient, and when appropriate, the informal caregiver. The stroke nurse held primary responsibility during the in-patient phase for understanding the patient’s experiences of the stroke in dialogue with the patient, integrating this with clinical findings, coordinating rehabilitation planning with the multidisciplinary team, and preparing discharge. This included coordination of contacts with primary health care, home help services, and the caregiver, as well as overall responsibility for discharge planning and continued rehabilitation.

Individual rehabilitation goals were formulated based on the patient’s context, history, strengths, and perceived needs, following a person-centred approach inspired by the Canadian Occupational Performance Measure [1]. Examples of goals included resuming everyday activities such as; shopping, household tasks, travelling independently, or managing finances.

At discharge, patients received an individualized rehabilitation schedule for the first week at home. The home based intervention comprised two to four weekly visits by physiotherapists and/or occupational therapists and one to two visits by the stroke nurse. The intervention focused on activity based training, problem solving strategies, adaptation to challenging situations, and supported practice to enhance confidence and safety.

Speech and language therapy was provided through outpatient services when needed. Throughout the intervention period, information and support were also provided to next of kin and home care services to facilitate ongoing support.

The intervention lasted for a maximum of four weeks after discharge, with termination jointly decided by the patient and the rehabilitation team. During this period, coordination was undertaken to ensure continuity of care with services responsible for ongoing rehabilitation after the VESD period. A more detailed description of the intervention is available in the GOTVED study protocol [2].

1. Law M, Baptiste S, McColl M, Opzoomer A, Polatajko H, Pollock N. The Canadian Occupational Performance Measure: An Outcome Measure for Occupational Therapy. Canadian journal of occupational therapy (1939). 1990;57(2):82-7. doi: 10.1177/000841749005700207.

2. Sunnerhagen KS, Danielsson A, Rafsten L, Björkdahl A, Axelsson ÅB, Nordin Å, et al. Gothenburg very early supported discharge study (GOTVED) NCT01622205: a block randomized trial with superiority design of very early supported discharge for patients with stroke. BMC Neurology. 2013;13(1):66. doi: 10.1186/1471-2377-13-66.
